# Immune determinants of Barrett’s progression to esophageal adenocarcinoma

**DOI:** 10.1172/jci.insight.143888

**Published:** 2021-01-11

**Authors:** Kiran H. Lagisetty, Dyke P. McEwen, Derek J. Nancarrow, Johnathon G. Schiebel, Daysha Ferrer-Torres, Dipankar Ray, Timothy L. Frankel, Jules Lin, Andrew C. Chang, Laura A. Kresty, David G. Beer

**Affiliations:** 1 Department of Surgery, Section of Thoracic Surgery, University of Michigan, Ann Arbor, Michigan, USA.; 2 VA Ann Arbor Health Care System, Ann Arbor, Michigan, USA.; 3 Department of Internal Medicine and; 4 Department of Radiation Oncology, University of Michigan, Ann Arbor, Michigan, USA.

**Keywords:** Immunology, Oncology, Cancer immunotherapy, Gastric cancer, T cells

## Abstract

Esophageal adenocarcinoma (EAC) develops from Barrett’s esophagus (BE), a chronic inflammatory state that can progress through a series of transformative dysplastic states before tumor development. While molecular and genetic changes of EAC tumors have been studied, immune microenvironment changes during Barrett’s progression to EAC remain poorly understood. In this study, we identify potential immunologic changes that can occur during BE-to-EAC progression. RNA sequencing (RNA-Seq) analysis on tissue samples from EAC patients undergoing surgical resection demonstrated that a subset of chemokines and cytokines, most notably *IL6* and *CXCL8*, increased during BE progression to EAC. xCell deconvolution analysis investigating immune cell population changes demonstrated that the largest changes in expression during BE progression occurred in M2 macrophages, pro–B cells, and eosinophils. Multiplex immunohistochemical staining of tissue microarrays showed increased immune cell populations during Barrett’s progression to high-grade dysplasia. In contrast, EAC tumor sections were relatively immune poor, with a rise in PD-L1 expression and loss of CD8^+^ T cells. These data demonstrate that the EAC microenvironment is characterized by poor cytotoxic effector cell infiltration and increased immune inhibitory signaling. These findings suggest an immunosuppressive microenvironment, highlighting the need for further studies to explore immune modulatory therapy in EAC.

## Introduction

The events by which Barrett’s esophagus (BE) develops into esophageal adenocarcinoma (EAC) are secondary to a series of transformative steps whereby BE is established in response to nonerosive reflux, followed by progression to low-grade dysplasia (LGD), high-grade dysplasia (HGD), and EAC. The histopathologic progression is accompanied by genomic and molecular changes, including loss of the tumor suppressive function of *TP53* (1*,* 2). The concerning consequence of BE is the 30-fold increased risk for developing EAC among these patients (3). Although much is known about the molecular and genomic changes that occur within the primary tumor, the interaction with the tumor microenvironment has only recently begun to be understood.

The local immune response appears to be a major component of the tumor microenvironment. Interactions among a combination of lymphoid cells (i.e., T cells, B cells, NK cells) and myeloid-derived cells (i.e., macrophages, neutrophils, eosinophils), as well as their secreted cytokines and chemokines, contribute to a dynamic flux between protumorigenic and antitumorigenic activity. Previous works have demonstrated the role of cytokines IL-6, CXCL8 (IL-8), TGF-β, and IL-17A in BE-to-EAC progression (4–8). In addition, work on the roles of specific immune cell subsets, such as Tregs, tumor-associated macrophages (TAM), and myeloid-derived suppressor cells (MDSC) demonstrate their ability to exert effects on tumor biology, including initiating metastasis and impacting the prognostic importance of these cell types in EAC patients (9–12). The ability to exploit the innate immune system to successfully treat late-stage cancer has been demonstrated through several landmark clinical trials targeting immune checkpoint inhibitors, including PD-1, PD-L1, and CTLA4 (13–15). However, efficacy of immune checkpoint inhibition in metastatic EAC has been mixed with response rates usually less than 20%, suggesting that there is more to be learned about the EAC immune microenvironment (16–21).

Previous immunohistochemically based studies have provided an initial understanding of the tumor immune microenvironment in esophageal cancer (22–27). However, many of these studies were performed primarily in mice and focused on esophageal squamous cell carcinomas. Further characterization of the local immune cell molecular landscape has been enhanced through gene expression data analysis using programs such as CIBERSORT (https://cibersort.stanford.edu) and xCell (https://xCell.ucsf.edu), which estimate comprehensive levels of immune cell subtypes (28–30). However, these techniques are limited by a lack of spatial context, marker protein levels, and direct cell counts. And while changes in cytokines, chemokines, and immune cell subsets have been studied in individual cohorts of BE or EAC patients, the changes have not been investigated in the context of tissue-specific changes within BE, LGD, HGD, and EAC.

To better understand the complex immunologic changes that accompany the dysplastic transformation of BE toward EAC, we applied a combination of immunohistochemical and gene sequencing techniques to understand the key cellular changes, along with changes in cytokine and chemokine expression. Using the program xCell (30), we performed a gene set enrichment with deconvolution approach to identify gene signatures of 64 different cell types, including prominent adaptive and innate immune cell types. We then sought to confirm these observations using multiplex IHC to characterize the expression of specific immune cell types. In addition, we analyzed individual cytokines, chemokines, and their associated receptors based on RNA-Seq expression profiles. The approaches presented here to analyze the data can be used both to identify new therapeutic targets specifically in the precancerous stages of BE transformation and to identify patients who may be candidates for immune modulating therapy such as immune checkpoint inhibition.

## Results

### Cytokine/chemokine profiling of Barrett’s progression to EAC using RNA-Seq.

**Figure 1:**
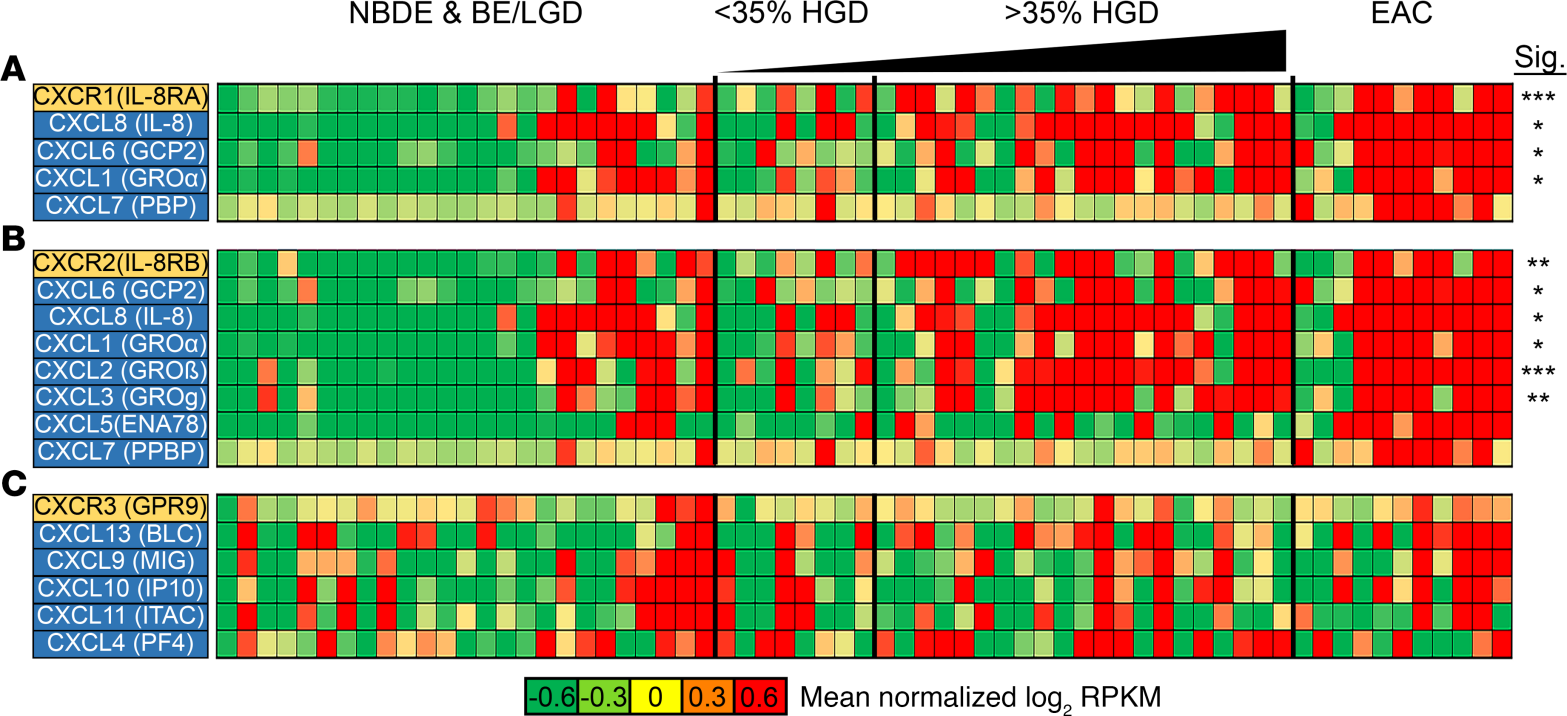
Changes in CXC family chemokine expression during progression from NDBE to EAC. RNA-Seq analysis was performed on samples from 65 direct-to-surgery EAC patients undergoing curative resection. Heatmaps showing gene expression changes in a subset of the CXC subfamily of chemokines. (**A**–**C**) Genes from the CXCR1 (**A**), CXCR2 (**B**), or CXCR3 (**C**) families are either upregulated (red) or downregulated (green) during progression from NDBE to EAC. *n* *=* 25 patients with NBDE or BE/LGD; *n* *=* 8 patients with < 35% HGD; *n* *=* 21 patients with > 35% HGD; *n* *=* 11 patients with EAC. *P < 0.05; **P < 0.01; ***P < 0.001; as determined by Welch’s 2-tailed *t* test and Wilcoxon rank-sum tests between BE/LGD versus HGD/EAC on log-transformed expression levels (log[RPKM + 1]).

**Figure 2:**
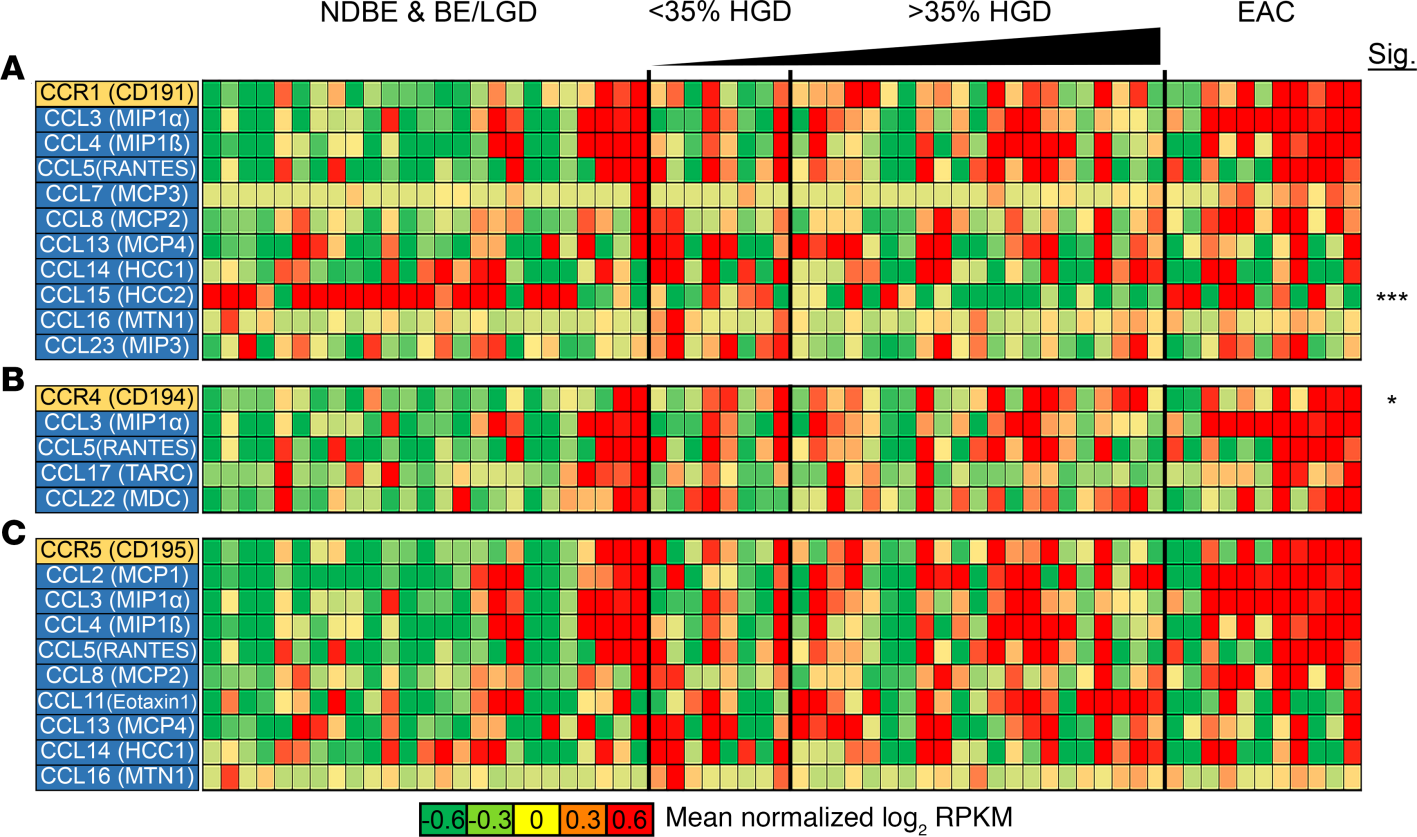
Changes in CC family chemokine expression during progression from NDBE to EAC. Heatmaps show changes in gene expression from a subset of the CC receptor family. (**A**–**C**) The CCR1 (**A**), CCR4 (**B**), and CCR5 (**C**) receptors and their associated ligands are shown for changes in gene expression during progression. *n* *=* 25 patients with NBDE or BE/LGD; *n* *=* 8 patients with < 35% HGD; *n* *=* 21 patients with >35% HGD; *n* *=* 11 patients with EAC. *P < 0.05; ***P < 0.001; as determined by Welch’s 2-tailed *t* test and Wilcoxon rank-sum tests between BE/LGD versus HGD/EAC on log-transformed expression levels (log[RPKM + 1]).

**Figure 3:**
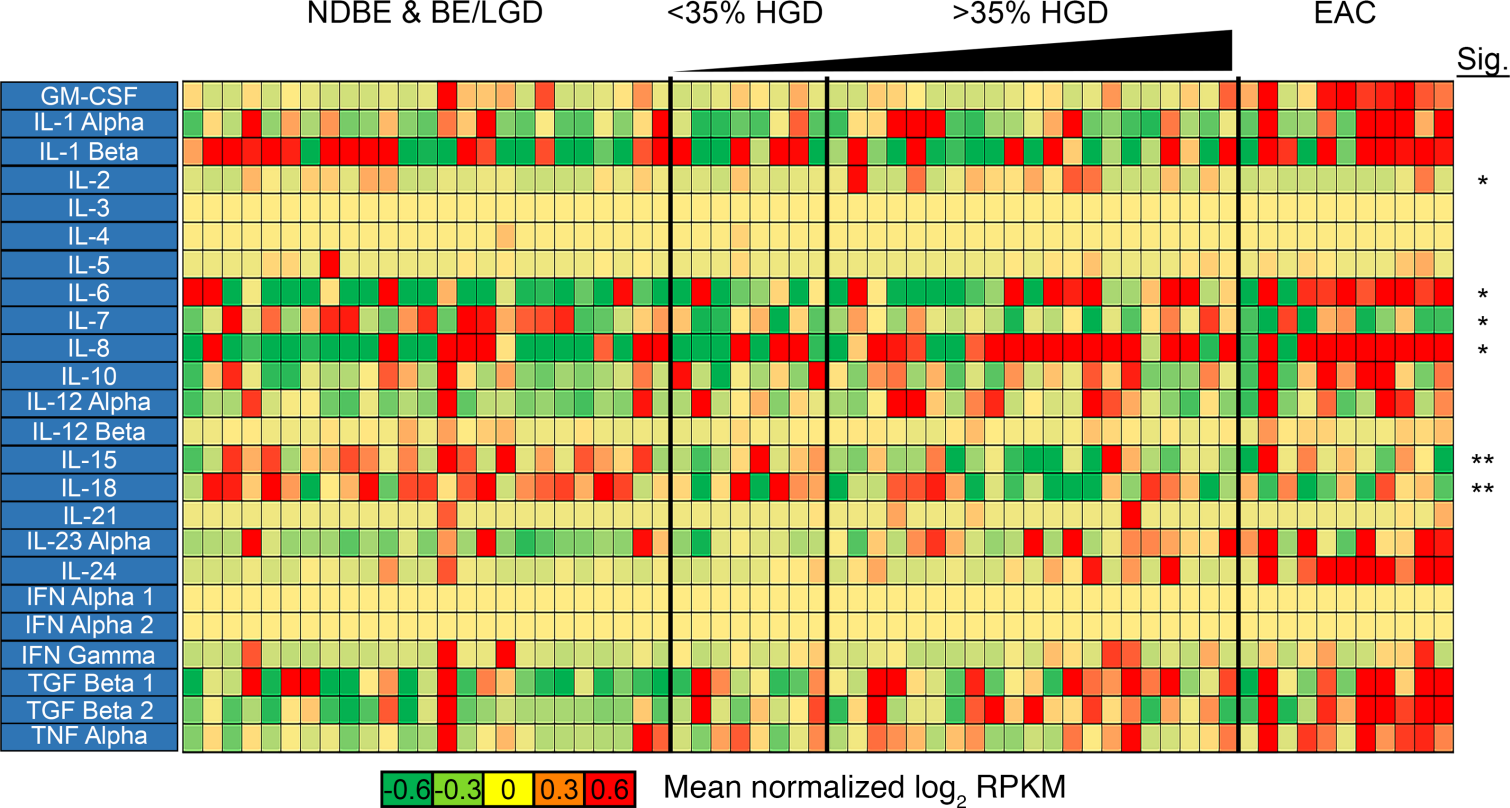
Cytokine expression changes during NDBE-to-EAC progression. Heatmap indicates changes in immune-related cytokine expression during progression. The most significant changes are in the IL gene family of cytokines. *n* *=* 25 patients with NBDE or BE/LGD; *n* *=* 8 patients with < 35% HGD; *n* *=* 21 patients with >35% HGD; *n* *=* 11 patients with EAC. *P < 0.05; **P < 0.01; as determined by Welch’s 2-tailed *t* test and Wilcoxon rank-sum tests between BE/LGD versus HGD/EAC on log-transformed expression levels (log[RPKM + 1]).

RNA-Seq was performed on a panel of 65 patient samples representing nondysplastic BE (NDBE) + BE/LGD (*n* *=* 25), HGD < 35% (*n* *=* 8), HGD > 35% (*n* *=* 21), and EAC (*n* *=* 11). Samples were verified by a pathologist, and HGD specimens were scored by percent of dysplasia present in the sample. Cytokine and chemokine expression analysis, along with their corresponding receptors, was performed as demonstrated in Figures 1, 2, and 3.

Chemokines receptors, such as *CXCR1* and *CXCR2*, were found to be consistently upregulated in a majority of EAC and HGD samples, compared with BE/LGD. In addition, their associated chemokines, including *CXCL6*, *CXCL8*, and *CXCL1*, showed significant upregulation in HGD/EAC (Figure 1). *IL6* and *CXCL8*, which have been shown to be involved in acute inflammation, were 3- to 4-fold higher in expression in 9 of 11 EAC samples compared with BE/LGD (Supplemental Tables 1 and 3; supplemental material available online with this article; https://doi.org/10.1172/jci.insight.143888DS1). Effector CD8^+^ T cell and NK cell receptor *CXCR3* was not significantly upregulated, nor were its respective chemokine targets, *CXCL9* or *CXCL10* (Figure 1 and Supplemental Table 1). These cytokines and receptor combinations have been demonstrated in immune cell activation. *CXCR4*, which typically has low expression in normal human tissues, trended toward increased expression in samples with increasing amounts of HGD and a majority of EAC samples, although it did not reach significance (Supplemental Figure 1). Its ligand, *CXCL12*, did not significantly change during progression (Supplemental Figure 1 and Supplemental Table 1). The B cell chemoattractant *CXCL13* was increased in a portion of samples (12 of 21 HGD and 7 of 11 EAC); however, this change was not significant.

The chemokine receptor *CCR1*, along with immune recruiting cytokines *CCL3*, *CCL5*, and *CCL7*, were all found to be upregulated in 8 of 11 EAC samples compared with HGD and LGD. However, comparing EAC/HGD with BE/LGD did not show a significant difference in either *CCR1* or many of its ligands, outside of *CCL15* (Figure 2). *CCL2*, which has been shown to be involved in macrophage and T cell recruitment, showed increased expression in 9 of 11 EAC samples, while its receptor, *CCR2*, was only overexpressed in 5 of 11 samples (Supplemental Figure 2 and Supplemental Table 2). *CCR5*, which has been implicated in metastasis, and its associated ligand, *CCL3*, trended toward upregulation in 7 of 11 EAC patients (Figure 2). Additional chemokine receptors, such as *CCR4*, *CCR6*, and *CCR7*, only showed a modest increase in subsets of HGD and EAC samples (Figure 2 and Supplemental Figure 2).

Interestingly, the largest fold-changes observed in gene expression during progression from NDBE to EAC were among the cytokine family and their associated receptors (Figure 3). Acute phase reactants *IL6* and *CXCL8* demonstrated the largest increase in expression. Both cytokines were significantly increased 4-fold in HGD/EAC samples compared with BE/LGD (Supplemental Table 3). Specifically, *CXCL8* was increased in 9 of 11 EAC samples and 15 of 21 HGD samples, where increased expression was associated with an increased percentage of HGD. In comparison, *IL6* was significantly increased in the same 9 of 11 EAC samples but only showed higher gene expression in 6 of 21 HGD samples (Figure 3). Immune-stimulating cytokines, such as *IL1B*, *IL2*, *IL4*, *IFNG*, *TNF*, and *TGFB1*, only showed modest increases in expression during BE progression to EAC (Figure 3, Supplemental Figure 3, and Supplemental Tables 3 and 5). The one exception was *IL24*, which showed no expression difference between BE/LGD and HGD but was upregulated in 9 of 11 EAC samples (Figure 3, Supplemental Figure 3, and Supplemental Tables 3 and 5). Interestingly, *IL7,* *IL15,* and *IL18* all showed modest, yet significant, decreases in expression in HGD/EAC compared with BE/LGD (Figure 3).

**Figure 4:**
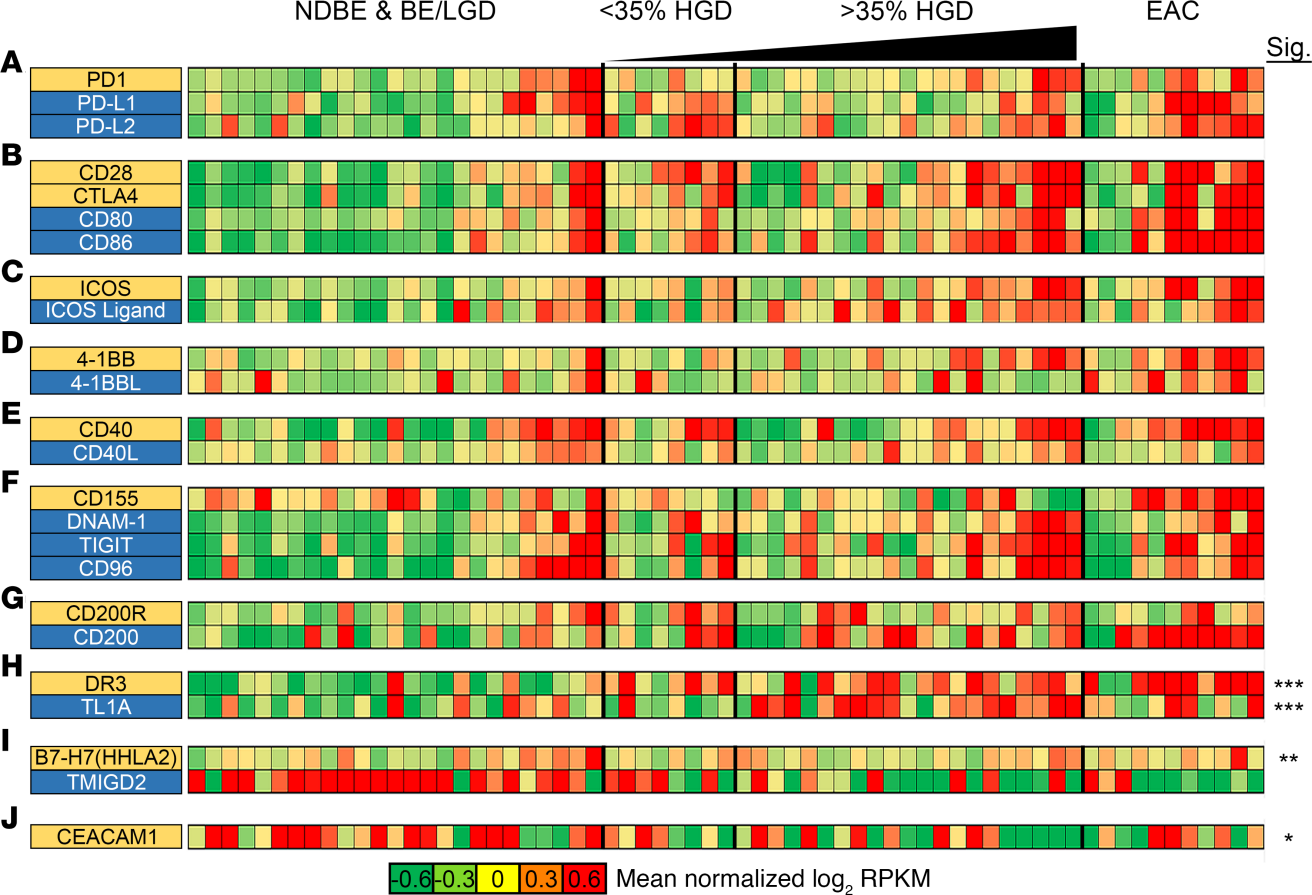
Expression changes in immune checkpoint marker genes during progression from NDBE to EAC. (**A**–**J**) Gene expression changes for the indicated immune checkpoint receptors and their associated ligands during progression. *n* *=* 25 patients with NBDE or BE/LGD; *n* *=* 8 patients with <35% HGD; *n* *=* 21 patients with >35% HGD; *n* *=* 11 patients with EAC. *P < 0.05; **P < 0.01; ***P < 0.001; as determined by Welch’s 2-tailed *t* test and Wilcoxon rank-sum tests between BE/LGD versus HGD/EAC on log-transformed expression levels (log[RPKM + 1]).

In addition to investigating changes in cytokine and chemokine expression, we analyzed changes in the expression of immune checkpoint pathways (Figure 4 and Supplemental Figure 4). In HGD, the patient samples exhibiting the highest immune checkpoint expression corresponded to the highest percentage of dysplasia. In cancer samples, elevated expression of immune checkpoint pathways was observed in roughly half of the EAC tissues (Figure 4 and Supplemental Table 4). While not significant, the general trend was that samples with increased expression of one immune checkpoint pathway tended to have increased expression in multiple checkpoint pathways. This includes both stimulating pathways such as *CD80/CD86* and *TNFRSF9* (4-1BB) as well as inhibitory pathways such as *PDCD1* (PD-1), *CD274* (PD-L1), *PDCD1LG2* (PD-L2), *CTLA4*, *ICOS*, and *TIGIT* (Figure 4). Interestingly, a member of the TNF receptor superfamily, *TNFRSF25* (death receptor 3; DR3), and its cognate ligand, *TNFSF15* (TL1A), were both significantly upregulated during progression from NDBE to EAC. Another immune-stimulating protein exhibiting expression differences independent of other pathways was *TMIGD2*. TIMGD2, in combination with its receptor, B7-H7, are involved in T cell proliferation and cytokine production. Our mRNA expression data suggest that this pathway was significantly downregulated during progression from BE to EAC.

### Immune cell expression using xCell gene analysis

**Figure 5:**
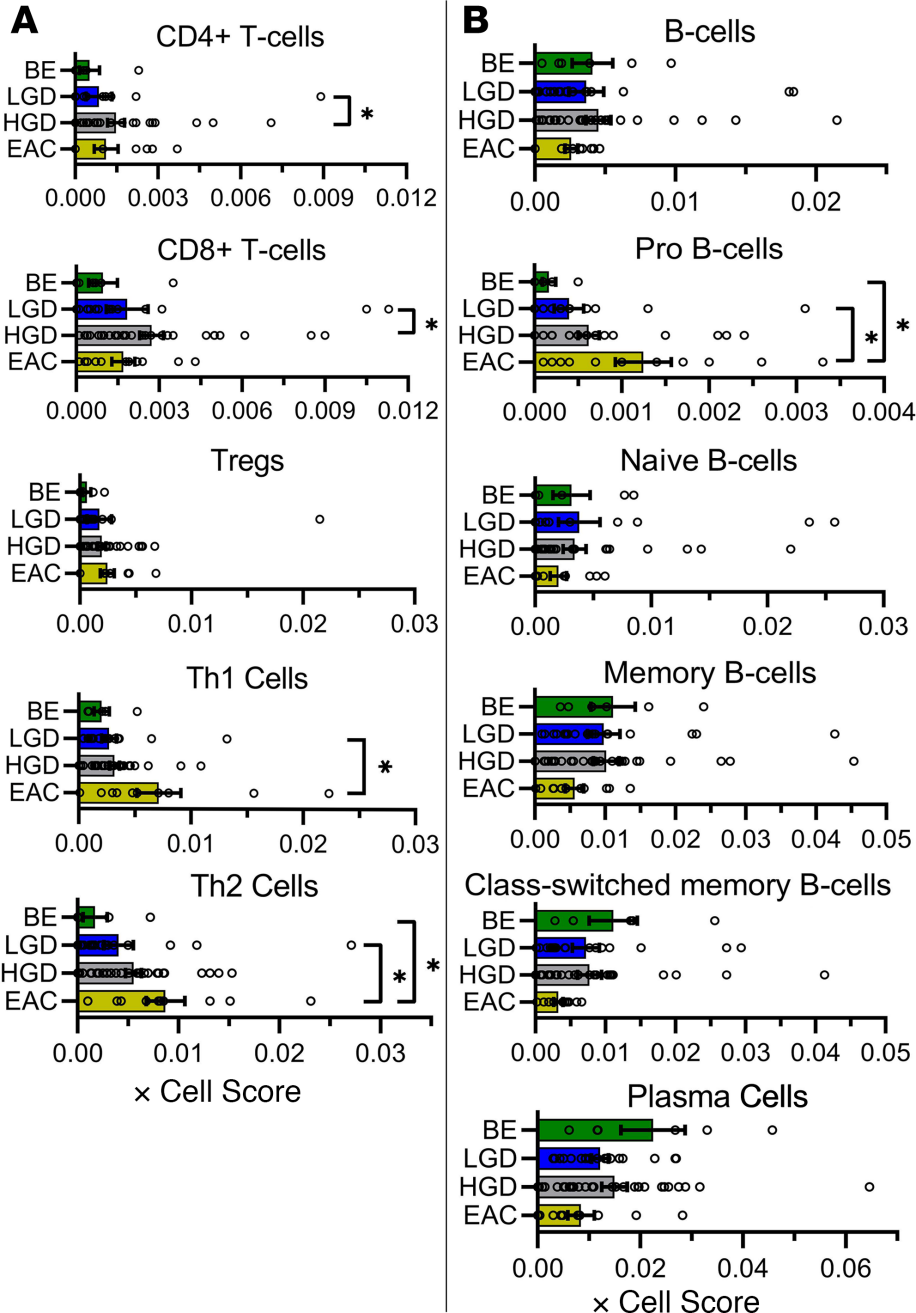
xCell analysis demonstrating changes in T cell and B cell lymphocyte gene signatures during progression from NDBE to EAC. xCell score obtained in silico using xCell for tissue samples from patients undergoing curative resection surgery. Histograms representing the mean value ± SEM are shown, along with individual data points. Changes in T cell (**A**) and B cell lymphocytes (**B**). *P < 0.05; as determined by nonparametric 1-way Kruskal-Wallis ANOVA with Dunn’s correction analysis.

**Figure 6:**
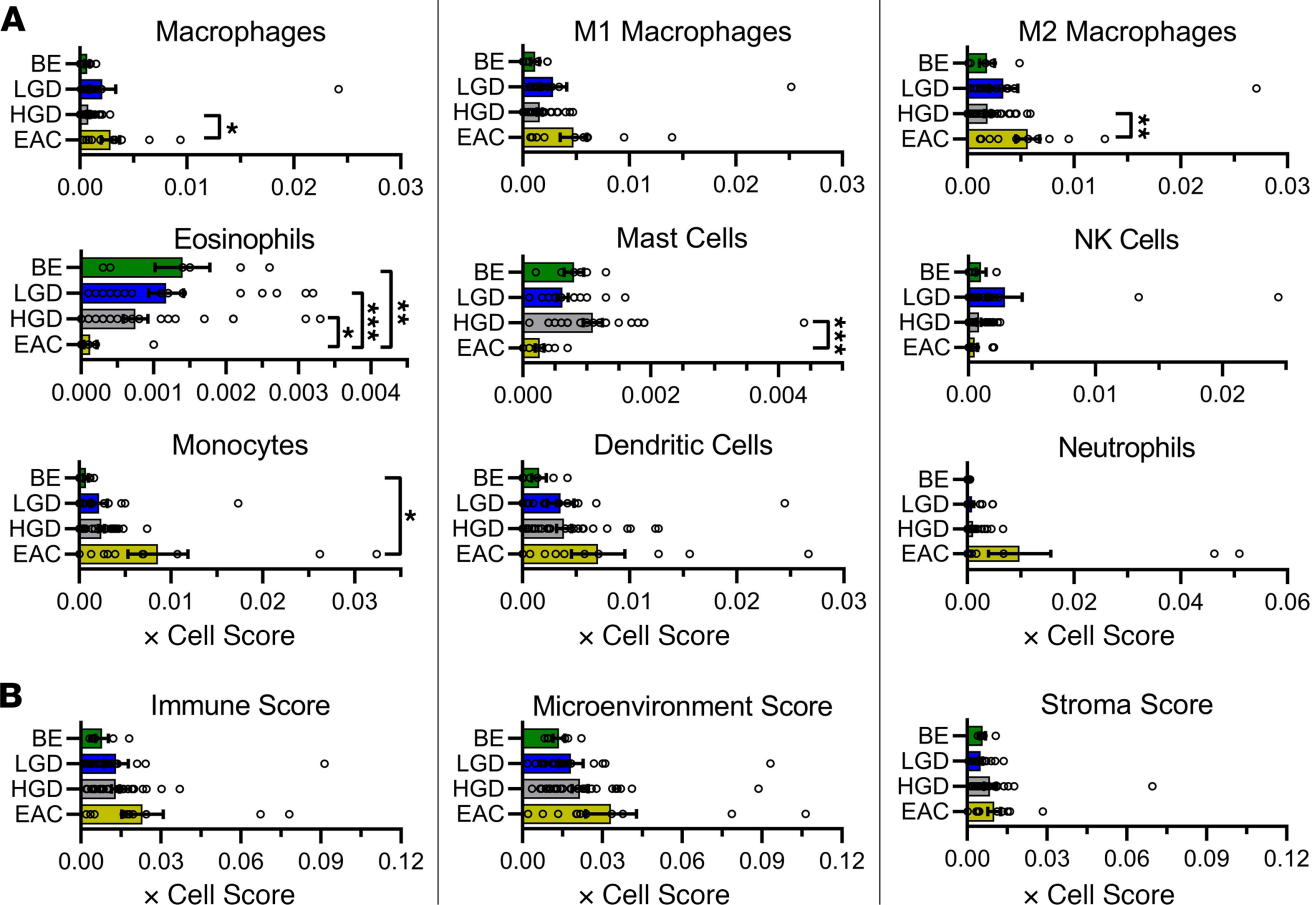
xCell analysis demonstrating changes in myeloid cell gene signatures and immune scores during progression from NDBE to EAC. xCell Score obtained in silico using xCell for tissue samples from patients undergoing curative resection surgery. Histograms representing the mean value ± SEM are shown along with individual data points. Changes in myeloid cells (**A**) and tissue scores (**B**). *P < 0.05; **P < 0.01; ***P < 0.001 as determined by nonparametric 1-way Kruskal-Wallis ANOVA with Dunn’s correction analysis.

In order to characterize changes of immune cell response during NDBE progression to EAC, we estimated immune cell fractions using the program xCell. xCell uses gene signatures for 64 cell types, including adaptive and innate immune cells, hematopoietic progenitors, epithelial cells, and extracellular matrix cells, which were derived from thousands of expression profiles. The complete analysis can be found in Figures 5 and 6 and in Supplemental Figures 5 and 6. Figures 5 and 6 represent immune cells and immune cell subsets. Although the immune score between NDBE, LGD, HGD, and EAC samples did not differ, there were variations noted in certain cell populations. Within T cells, there were no significant increases in CD4, CD8, or Treg populations or subpopulations. However, Th1 and Th2 cells were generally upregulated in EAC, where Th1 cells were increased in EAC compared with LGD and Th2 cells were increased compared with both NDBE and LGD populations (Figure 5A). The myeloid cell populations appeared stable within the overall macrophage population. However, when broken down between M1 and M2 macrophages, differences were detected (Figure 6A). While not significant, there appear to be 2 distinct M1 subpopulations, high- and low-expressing, within the EAC samples. M2 macrophages were found to be significantly increased in EAC samples only when compared with HGD samples (Figure 6A). Interestingly, eosinophils were significantly decreased in EAC samples when compared with each of BE, LGD, and HGD sample groups (Figure 6A). Conversely, pro–B cells were significantly increased in EAC samples when compared with BE and LGD (Figure 5B). Pro–B cells are notable for the stage of B cell differentiation in which heavy chain rearrangement occurs. The remaining B cell populations demonstrated no significant differences.

### Validation of selected immune cell populations using multiplex IHC

**Figure 7:**
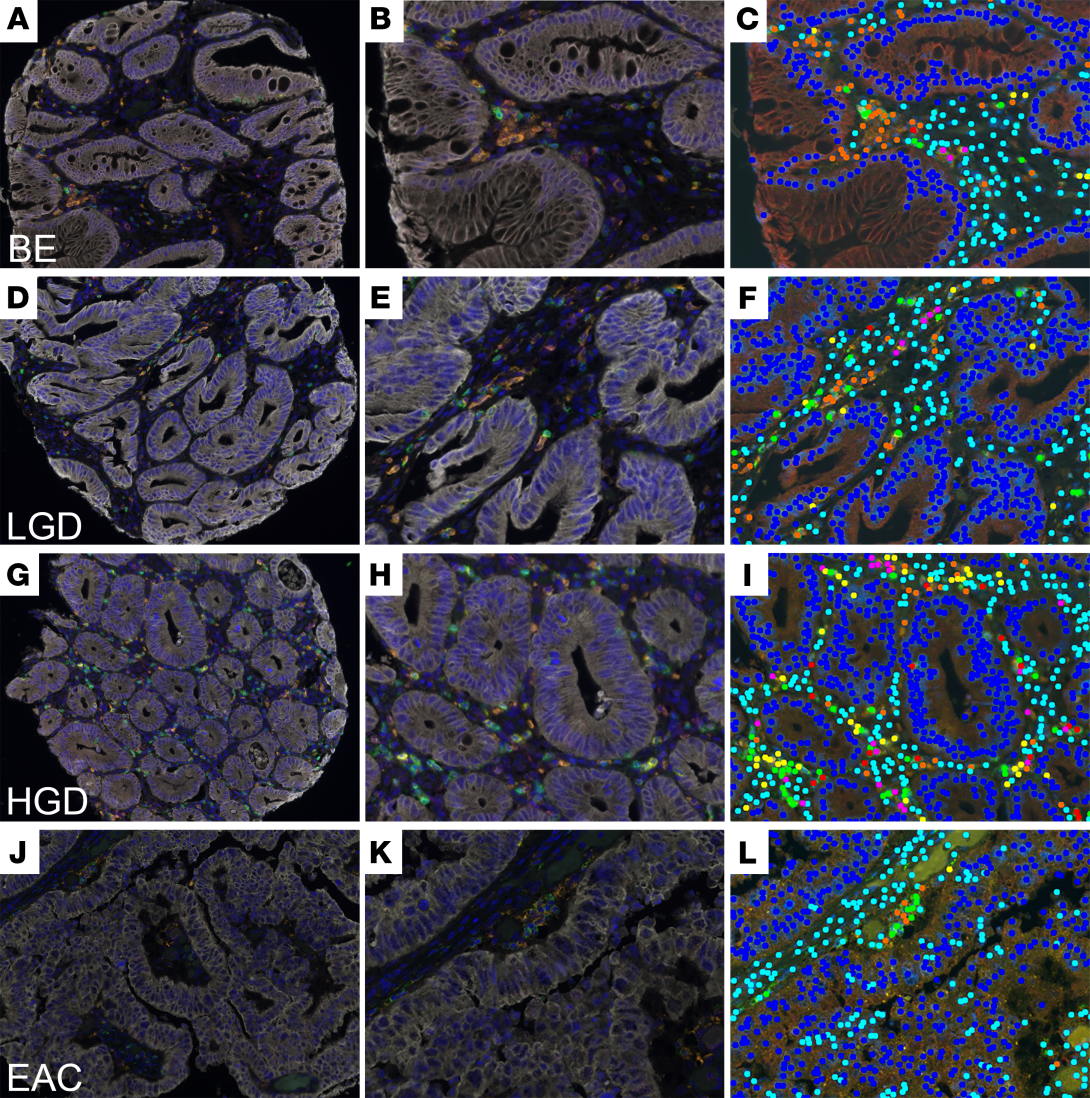
Multiplex immunohistochemical analysis of tissue sections from NDBE, LGD, HGD, and EAC tissue microarrays. Multiplex IHC staining (CD3, green; CD8, yellow; CD163, orange; FoxP3, red; PD-L1, magenta; and PanCK, white) in an esophageal TMA. Total original magnification, 20×. Sections were counterstained with DAPI to visualize nuclei. (**A**, **D**, **G**, and **J**) Composite images from BE, LGD, HGD, and EAC showing immune cell spatial resolution within each core. (**B**, **E**, **H**, and **K**) Magnification of the composite images showing detailed immune cell staining. (**C**, **F**, **I**, and **L**) Identification of immune cell phenotypes within the images using InForm software algorithms.

**Figure 8:**
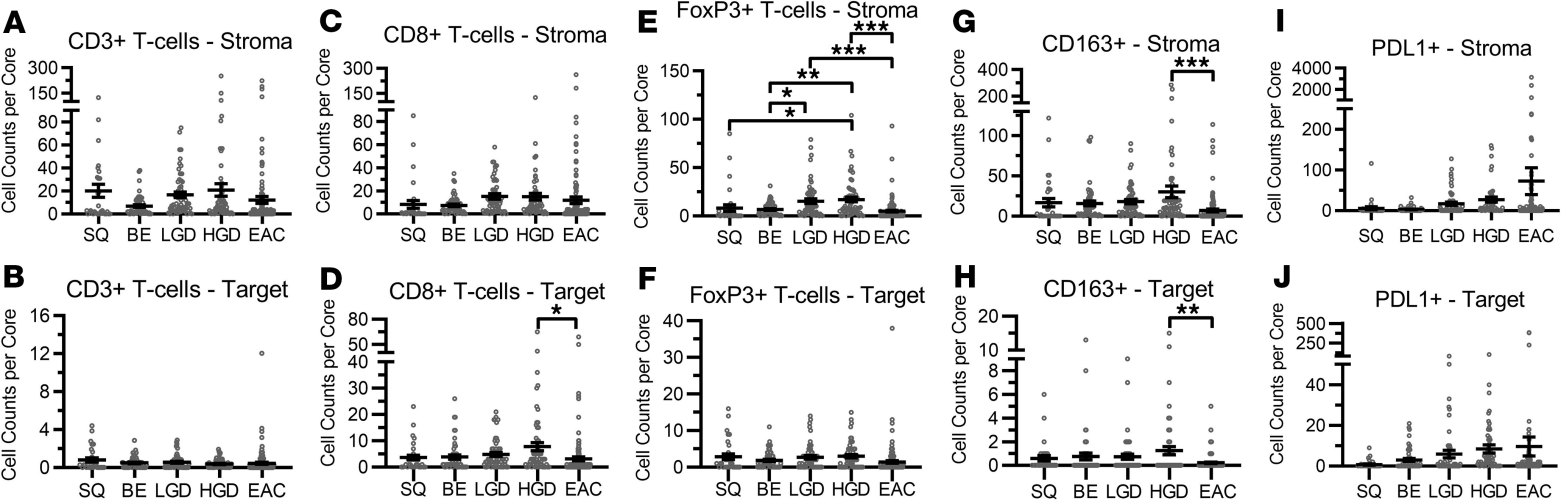
Spatial analysis of immune cell populations in EAC Progression. (**A**, **C**, **E**, **G**, and **I**) Quantitation of the number of the respective immune cells per core localized in the stromal tissue of each section. (**B**, **D**, **F**, **H**, and **J**) Quantitation of the number of respective immune cells per core localized in the target PanCK^+^ (epithelial) tissue. SQ, squamous epithelium (*n* *=* 32); BE, nondysplastic BE (*n* *=* 54); LGD, low-grade dysplasia (*n* *=* 58); HGD, high-grade dysplasia (*n* *=* 60); EAC, esophageal adenocarcinoma (*n* *=* 125). Data are presented as mean ± SEM. *P < 0.05; **P < 0.01; ***P < 0.001 as determined by 1-way ANOVA with Tukey correction for multiple comparisons.

We attempted validation of key findings for several cell types predicted through the above analyses by simultaneously evaluating protein expression of select cell surface markers: CD3 (T cells), CD8 (cytotoxic T cells), CD163 (macrophages), FoxP3 (Tregs), PD-L1, and DAPI (Figures 7 and 8). Two esophageal TMAs containing tissue cores from 54 BE sections, 58 LGD sections, 60 HGD sections, and 125 EAC sections were stained for all markers (Figure 7). We imaged at least 75% of each TMA core and analyzed the localization of each cell population within the stroma and adjacent to the esophageal epithelial cells (target) separately (Figure 8). Within the stroma, the most notable change was in the Treg (CD3^+^FoxP3^+^) population. These cells demonstrated a significant increase from BE to LGD and, subsequently, to HGD (Figure 8E). There was then a statistically significant decline of Tregs between HGD and EAC, as determined by 1-way ANOVA analysis with multiple comparisons (Figure 8E). The other notable decline was loss of CD163 cells from HGD and EAC (Figure 8G). When immune cell counts were analyzed in areas adjacent to target tissue, we found disparate results, especially within the Treg population. There were no significant changes across all tissues, based on histopathology, in terms of Treg counts (Figure 8F). In contrast to the stroma, there was a gradual rise, although not significant, of CD3^+^CD8^+^ cytotoxic T cells across BE tissue groups, NDBE to HGD (Figure 8D). There was, however, a significant decline in CD3^+^CD8^+^ cytotoxic T cells in EAC samples compared with HGD (Figure 8C). CD163^+^ macrophages were also found to be significantly decreased at the target tissue in EAC, similar to what we observed in the stroma (Figure 8H). When we evaluated the immune checkpoint PD-L1, we saw increased expression in both the stroma fraction and the target tissue (Figure 8, I and J). This upward trend, while not significant, was consistent with our RNA-Seq analysis.

## Discussion

These data represent a comprehensive assessment of the immunologic changes that accompany Barrett’s progression to EAC. We utilized multiple approaches and platforms to describe immune-linked genomic and protein level changes that occur during progression of BE. Transcriptomic analysis was utilized to describe message-level changes of a panel of important chemokines and cytokines. We performed deconvolution analysis with xCell to predict immune cell populations and their subtypes. Furthermore, multiplex IHC was employed to explore the contextual relationship between specific immune cells based on esophageal pathology, as well as between the target epithelium and stroma.

There are subtle differences as BE progresses from LGD to HGD and EAC, demonstrating that, in a subset of patients, immune changes associated with cancer formation occur in a stepwise fashion as opposed to abrupt changes in cellular composition. Transcriptome analysis demonstrated a significant increase in chemokines such as *IL6* and *CXCL8*, along with the CXCL8 receptors, *CXCR1* and *CXCR2*. Additionally, immune cell phenotyping via RNA-Seq and the genomic cellular analysis tool xCell provided evidence for a linear increase in Th1, Th2, Tγδ, and pro–B cell populations in EAC compared with precancerous histologies (BE, LGD, HGD). xCell analysis demonstrated a linear increase in M1 and M2 macrophages between HGD and EAC. Conversely, most T cell populations demonstrated no significant change, with a relatively flat change when measured at the transcriptomic level. Next, to gain spatial understanding of immune-linked changes, multiplex IHC analysis was performed on a TMA panel representing BE, LGD, HGD, and EAC. Multiplex IHC analysis demonstrated a significant decrease in the number of T cells and Tregs in EAC samples compared with HGD. Immune cell populations tended to increase in a stepwise fashion from BE to LGD to HGD, followed by a decline in all evaluated immune cell populations in EAC tissues. This decline in immune cell populations coincided with increased PD-L1 expression. Recent evidence suggests that a rise in PD-L1 in both tumor cells and tumor immune cells was associated with prolonged survival in EAC (31). Taken together, it appears that Barrett’s progression to LGD and HGD increases immune cell infiltration across many different cell types. The transition to EAC, however, is characterized by loss of infiltrating immune cells, which may be explained in part by the dual role of the cytokines IL-6 and IL-8, which initially provide an inflammatory, antitumorigenic stimulation and subsequently produce protumorigenic signals during the transition from HGD to EAC. Both the transcriptome analysis and protein characterization demonstrated that certain subgroups of tumors tend to overexpress multiple cytokines, chemokines, and immune cells that may represent a subset of patients who might respond favorably to immune modulating therapies.

The role of IL-8 in Barrett’s progression was recently described in a paper by Münch et al., which demonstrated that a high-fat diet induces IL-8 secretion and plays a role in accelerated dysplastic growth in a mouse model of BE (32). This model also demonstrated increased recruitment of immature myeloid cells and neutrophils. In our study, we demonstrated that the largest increase in *CXCL8* expression occurs with higher percentages of HGD and most EAC samples (Figures 1 and 3). xCell analysis further substantiated the role of IL-8 in EAC development, with increased numbers of macrophages and neutrophils documented in EAC tissues compared with premalignancy samples (Figure 6). Of note, the significant increase in macrophages was primarily within the M2 macrophage phenotype, which is typically thought of as a protumorigenic cell type. This further suggests that the role of IL-8 is dual purpose in these tissue microenvironments, with an initial proinflammatory, antitumorigenic state followed by a protumorigenic role in HGD and EAC. The initial increase in IL-8 results in chemotaxis of neutrophils, which in turn can promote the release of neutrophil extracellular traps leading to the elimination of microbes, as well as promote endothelial cell proliferation, survival, and migration (33, 34). This initial inflammatory, antitumorigenic role may subsequently be switched to a more protumorigenic role once a critical amount of HGD has occurred. The role of IL-8 in EAC is not unique, as it has been detected in multiple cancer types, including gastric, lung, melanoma, colon, ovarian, and prostate cancers (35–40). A possible mechanism of IL-8–induced carcinogenesis is through the recruitment of immunosuppressive cells, such as MDSCs, into the tumor immune microenvironment. A previous study in a transgenic mouse model carrying the human *CXCL8* gene demonstrated accelerated growth of gastric and colon cancers through the recruitment of MDSCs (41). This finding represents a possible precancerous intervention using IL-8 receptor antagonists such as Repertaxin. A preclinical gastric cancer model using Repertaxin has shown enhanced efficacy of 5-fluorouracil, with decreased cell proliferation, migration, and invasion (42). Another study in breast cancer demonstrated that cytotoxic chemotherapy resulted in secretion of IL-8, subsequently stimulating adjacent cancer stem cells (43). In the latter study, Repertaxin specifically targeted cancer stem cells to reduce tumor growth and metastases. These data have provided the basis for an ongoing clinical trial targeting IL-8 signaling in patients with metastatic breast cancer (NCT02001974; https://clinicaltrials.gov/ct2/show/NCT02001974).

In addition to increased IL-8 and IL-6 expression, our data reveal a significant, albeit modest, decrease in 3 other IL family members, IL-7, IL-15, and IL-18. Interestingly, all 3 of these ILs have been shown to be involved aspects of lymphocyte proliferation, maintaining cytotoxic T cell levels, and promoting a proinflammatory, antitumor environment (44–49). IL-7 has been shown to promote the proliferation and survival of lymphocyte cell types, including T cells, B cells, and NK cells (44, 45). Furthermore, IL-7, in conjunction with hepatocyte growth factor, stimulates pro–B cells to differentiate into mature, functional B cell populations (44). The decrease in *IL7* expression observed in our studies could help explain the increase in pro–B cells described in our xCell analysis (Figure 5). Within the tumor microenvironment, IL-15 has been shown to maintain NK cell homeostasis and promote T cell proliferation (46, 47). In addition, IL-15 can promote the antitumor activity of CD8^+^ cytotoxic T cells in preclinical models. When given to mice, either exogenously or through transgenic expression in adoptively transferred T cells, IL-15 enhanced the in vivo function of CD8^+^ cytotoxic T cells (46). The decrease in expression in our RNA-Seq data supports that IL-15 may play a role in the loss of CD8^+^ T cells that we describe in both the stroma and target of EAC tumor tissues observed in our multiplex IHC results (Figure 8, C and D). Finally, IL-18 is a proinflammatory cytokine that promotes B cell survival and, in the presence of either IL-15 or IL-12, stimulates T cells, B cells, and NK cells to secrete IFN-γ, effectively enhancing antitumor cytotoxicity (48, 49). A loss of IL-18 could decrease the ability of effector cells to secrete IFN-γ and promote tumor cell survival. Taken together, the loss of IL-7, IL-15, and IL-18 may be playing a role in the decreased numbers of lymphocytes in the tumor microenvironment, which supports a protumorigenic environment.

Our xCell analysis uncovered 2 cell populations, eosinophils and pro–B cells, not previously well described as mediators of BE progression to EAC. Eosinophils significantly decline in expression across all stages of dysplasia and EAC progression (Figure 6A). Previous population-based studies have demonstrated a link between eosinophilic esophagitis, a condition characterized by high infiltration of eosinophils and lack of progression to esophageal cancer (50–52). The fact that no patients went on to develop esophageal cancer may be secondary to the relatively new nature of the diagnosis. The importance of CCR3 in eosinophil activation was shown in a study by Heath et al., which demonstrated over 95% of the response to eosinophil ligands were mediated through the CCR3 receptor (53). In the context of EAC, the mechanism of eosinophilic loss is not well understood. A review of study samples demonstrates that the eosinophil receptor, CCR3, is neither up- nor downregulated in Barrett’s progression. However, in our data, eosinophil-stimulating cytokines *CCL4* and *CCL5* both demonstrated upregulation in EAC, suggesting that the lack of CCR3 upregulation may result in a lack of eosinophil recruitment (Supplemental Figure 2). Previous reports on the role of eosinophils in carcinogenesis have demonstrated increased tumorgenicity in eosinophil-deficient mice, as well as improved survival in patients with esophageal squamous cell cancer with increased eosinophil counts (50, 54). These data suggest that eosinophils may provide an important role in immune surveillance, and the loss of these immune cells may contribute to progression toward EAC. Understanding the mechanism of loss of eosinophils may represent an important mechanism underlying Barrett’s progression toward dysplasia and cancer.

There is a growing body of literature describing the role of B cells in tumorigenesis. B cells are thought to interact with T cells by secretion of stimulating chemokines/cytokines such as IL-2, IL-4, IFN-γ, and TNF-α (55, 56). B cells also differentiate to plasma cells and are thought to directly secret tumor-specific antibodies. A recent systematic review of 69 studies demonstrated that tumor-infiltrating B cells had mostly a positive or neutral prognostic effect (57). In those studies focused on esophageal cancer, 1 study demonstrated a positive prognostic effect, with the other being neutral (58, 59). The limitation of these studies is that, often, only a single B cell marker was utilized; therefore, the specific lineage of B cells was not identified. Our data show an increase in a specific subset of B cells, the pro–B cell, which has not been studied in the context of EAC or other epithelial cell cancer development scenarios. The lower representation of more mature B cell populations suggests either an arrest at the pro–B cell stage, statistical noise incurred during cell fraction calculation in the xCell program, or insufficient analytic material.

In our attempts to link the findings from the xCell gene analysis to protein level immune changes, we performed multiplex IHC to identify the number and location of CD8^+^ T cells, Tregs, and macrophages. Differentiating these immune cells into stromal localization versus target epithelium demonstrate how the loss of CD8^+^ T cells was most dramatic at the epithelial cell level, suggesting little epithelial cell–CD8^+^ T cell engagement. Furthermore, after a rise of Tregs and macrophages from Barrett’s progression to HGD, there is an abrupt reduction in Treg number in EAC, suggesting that a loss of immune cells contributes to EAC development. One potential reason for this loss is the increasing rise of the expression of PD-L1. PD-L1 has been shown to cause T cell apoptosis and suppress antitumor immunity (60, 61). Both our IHC and RNA-Seq data show a stepwise rise in PD-L1 expression. The increased PD-L1 expression, however, is not seen in all samples, and our transcriptome analysis supports that tissues with an increase in one immune checkpoint pathway tend to have an increase in multiple immune checkpoint pathways. This suggests that these individuals may benefit from immunomodulatory therapy, such as anti–PD-1, anti–PD-L1, or anti-CTLA4 therapy. Future analysis will be focused on evaluating the location of PD-L1 expression and spatial analysis of PD-L1 cells to T cells and macrophages. In addition to the rise in PD-L1 expression, there is a concomitant loss of CD8^+^ T cells within the target tissue. This loss may also be explained by the rise of gene related to anergy in lymphocytes (GRAIL) within the T cell (62). Our previous work on GRAIL has demonstrated the presence of 2 isoforms, one of which has been shown to stabilize mutant p53. A study by Nurieva et al. demonstrated how GRAIL contributes to the breakdown of CD3 (63). This dual role of GRAIL may offer insight into how BE is eventually able to avoid immune surveillance during its progression to EAC. In addition to PD-L1, transcriptome analysis demonstrated a significant rise in the DR3/TL1A and B7-H7/TMIGD2 pathways.

TL1A belongs to the TNF superfamily of proteins and is typically found on activated T cells, macrophages, monocytes, and DCs. Its receptor DR3 is typically restricted to innate CD4^+^ T cells, CD8^+^ T cells, and B cells. The DR3/TL1A pathway has been associated with proinflammatory conditions, specifically acting as a costimulatory pathway of T cells to increase production of IL-2 (64, 65). This has been well described in inflammatory bowel disease conditions such as Crohn’s disease and ulcerative colitis (66, 67). Although we see very little increase in T cell populations in Barrett’s progression, a potential role for this pathway is Treg stimulation and proliferation. Stimulation of the TL1A/DR3 pathway in murine models has been shown to cause Treg proliferation and protect against inflammation (68). This pathway could explain the rise we see in stromal Tregs during Barrett’s progression.

The other checkpoint pathway found to be increased was the B7-H7/TMIGD2 pathway. B7-H7 is typically expressed on monocytes and macrophages, and TMIGD2 is typically expressed on naive T cells and NK cells. TMIGD2 stimulation was shown to inhibit T cell proliferation and cytokine production of IFN-γ and TNF-α (69). In addition, Janakiram et al. demonstrated that B7-H7 is overexpressed in esophageal cancer, as well as liver, bladder, colon, prostate, and kidney cancers (70). Given the poor results of immune checkpoint therapy in esophageal cancer, this may provide an additional pathway for targeted immune checkpoint therapy.

We used several different tools to describe the changes that occur during Barrett’s progression to EAC; however, each has its own limitations. RNA-Seq data of individual chemokines and cytokines may not accurately reflect the true level of these cytokines, and this type of analysis does not allow for evaluation of which cells are responsible for their secretion. xCell, although a valuable prediction tool, is subject to statistical noise and may over- or underestimate true expression of cell types within tissues. Multiplex IHC, while a useful tool in combination with xCell, is also limited by the number of antibodies that can be multiplexed at a given time. The expression of CD163^+^ in our multiplex IHC and discordance in our xCell analysis is an example of this limitation, where a significant decline in these macrophages was seen both in the stroma, as well as within the target tissue; however, it was suggested to be increased by xCell analysis. This may reflect differences in mRNA expression versus steady-state protein levels for this gene. Additional discordance was seen in the expression of Tregs, where there was no difference noted during sequencing analysis; however, a significant decline was noted in Tregs within the stroma on multiplex IHC analysis. This further demonstrates the importance of spatial localization, since Tregs are not significantly changed at the target tissue, which may underlie the findings on xCell analysis.

Future studies will need to be performed at the single cell level to validate many of the findings of our analysis. Single cell sequencing offers the unique ability to characterize multiple types of cell subsets and analysis at the message level of individual cells. This is still limited by lack of context, which multiplex IHC provides, but these techniques can be used in combination to validate findings.

In conclusion, this work demonstrates some of the key immunologic changes that occur during Barrett’s transformation to EAC. We were able to demonstrate the increased expression of inflammatory cytokines *IL6* and *CXCL8*, as well as their associated receptors *CXCR1/CXCR2*. T cell costimulatory pathways TL1A-DR3 and B7-H7–TMIGD2 were found to be significantly upregulated during Barrett’s progression. We also demonstrated the increased expression of M2 macrophages and uncovered how EAC is associated with a loss of eosinophils and the rise of pro–B cells. Multiplex staining demonstrated a stepwise rise in PD-L1 with concomitant loss of CD8^+^ T cells, Tregs, and macrophages during cancer formation. Taken together, these data offer several future study directions and offer potential therapeutic interventions based on existing therapies.

## Methods

### Patients.

For RNA-Seq analysis, tissue samples were collected from 65 chemo-naive patients undergoing curative resection for EAC at the University of Michigan Health System. For single immunohistochemical and multiplex immunohistochemical analysis, esophageal tissues were collected from a separate cohort of 209 patients diagnosed with HGD or EAC and undergoing curative cancer surgery at the University of Michigan Health System.

### RNA isolation and processing.

Patient tissues used for RNA-Seq analysis were isolated and handled, including histopathologic characterization for the percentage of dysplasia, as previously described (69). Briefly, all samples were first sectioned by cryostat and H&E stained to identify the optimal regions to be used for RNA isolation. The regions chosen were absent of any signs of necrosis, without extensive inflammation, and containing at least 70% tumor content. Stage 1–3 tumors were used, and none showed any signs of extensive ulceration. All BE, LGD, or HGD samples were obtained from patients presenting with either HGD or EAC and were acquired from a region within 6 cm of the tumor. Total RNA was isolated from these tissues by column purification using the miRNeasy Mini Kit as per manufacturer instruction (Qiagen). RNA purity and integrity were determined by a 2100 Bioanalyzer (Agilent Technologies).

### Analysis of changes in immune markers using RNA-Seq data.

Sample mRNA with RIN scores > 7.0 were used, and paired-end sequence analysis of 120 million 100-bp reads per lane was performed using Illumina sequencers at the University of Michigan DNA Sequencing Core Facility. Sequence alignment and analysis were performed as previously described (69). Fold-change of expression levels between BE and LGD versus HGD and EAC was summarized as the ratio of the median expression levels between the 2 groups. To investigate changes in immune cell populations during progression from BE to EAC, xCell analysis was performed on our RNA-Seq data set (*n* *=* 65 patients). xCell is a computational method used to investigate changes in cell composition based on bulk transcriptomic profiles (71). We generated cell type abundance scores using the standard 64 immune cell type signatures that have been previously created (30).

### Preparation of esophageal tissue microarrays.

BE, LGD, HGD, and EAC samples from 209 patients undergoing curative resection were collected, formalin fixed to preserve tissue architecture, and paraffin embedded for sectioning. Similar to RNA-Seq tissues, all BE, LGD, and HGD tissues were acquired from patients presenting with HGD or EAC and were acquired from a region within 6 cm of the tumor. H&E-stained sections prepared by the University of Michigan Pathology Department were used to confirm disease pathology. To create the tissue microarray (TMA), regions of the original tissue block were identified as areas of interest according to tissue pathology. From these regions, 2–3 cores were made for each patient’s tissue sample. Once generated, the TMA was sectioned and baked at 50°C for 24 hours to adhere the tissue sections.

### Multiplex IHC.

Multiplex IHC was performed using the Opal 7 Solid Tumor Immunology Kit (Perkin Elmer) according to manufacturer’s instruction, as previously described (72). TMA slides composed of esophageal, columnar-derived dysplasia, and EAC were sectioned and baked 60°C for 1 hour. Slides were deparaffinized with 3 changes of 100% xylene, followed by rehydration in a series of graded ethanol to distilled water. Slides were then dipped in neutral buffered formalin to increase tissue section adherence prior to the first antigen retrieval (AR) step. Primary antibody sources and dilutions are listed in Supplemental Methods. Slides were counterstained with DAPI to visualize nuclei prior to mounting using ProLong Diamond (Thermo Fisher Scientific).

### Visualization and quantitation of multiplex IHC slides.

Slides were imaged using a Mantra Quantitative Pathology Imaging System (QPIS). One to 2 images per core were acquired using 20× magnification. Filters for DAPI, CY3, CY5, CY7, Texas Red, and Qdot were applied to each image set for each core. To spectrally unmix and quantitate the individual fluorescent channels, a multispectral library was generated on the Mantra QPIS system, which was used to unmix the image and identify specific staining for each antigen. Image files created by Mantra were analyzed using InForm 2.2.1 image analysis software, as previously described (72). Background autofluorescence, defined as a signal from unstained esophagus tissue, was subtracted per image. Each cell nucleus was identified using spectral DAPI, and epithelial target tissue areas (Pan Cytokeratin–positive) were segmented. Areas of tissue negative for Pan Cytokeratin were categorized as stroma. Fluorescent intensities above background were determined for each marker and used to identify positive cells based on their staining profile. The number of CD3^+^, CD8^+^, CD163^+^, FoxP3^+^, and PD-L1^+^ cells were identified and segmented into the different tissue compartments (epithelial versus stroma). For quantitation, the TMAs were pathologically scored, and the tissue type was then recorded (S, normal squamous; G, gastric gland; BE, BE without dysplasia; LGD, BE with LGD; HGD, BE with HGD; EAC, cancer). Target tissue was defined as the pathologist-diagnosed tissue type within each individual core (BE, LGD, HGD, or EAC).

### Statistics.

For multiplex IHC analysis, all graphs were generated, and data were analyzed using Graph Pad Prism v 7.00. Statistical analysis of cell count data for each immunologic marker was performed using 1-way ANOVA followed by Tukey’s multiple-comparisons test to determine significant differences among the different cell types. For xCell analysis, data for each cell type were graphed using GraphPad Prism. Statistical significance was assessed using a nonparametric Kruskal-Wallis test for multiple comparisons with Dunn’s correction for statistical hypothesis testing. Welch’s 2-tailed *t* test and Wilcoxon rank-sum tests were used where indicated in figure legends. *P* < 0.05 was considered statistically significant. All histograms are presented as the mean value ± SEM.

### Study approval.

All patients provided informed consent, and all experimental protocols were approved by the University of Michigan IRB and Ethics Committee. The methods were carried out in accordance with approved guidelines.

## Author contributions

KHL was involved in developing the study concept and experimental design, developing methods, providing sample material and clinical information, interpreting data, supervising the study, preparing and revising the manuscript. DPM developed the study concept and experimental design, performed experiments, developed methodology, analyzed and interpreted data, and prepared and revised the manuscript. DJN helped with experimental design and study concept, method development, statistical analysis, and manuscript revision. JBS designed and performed experiments, analyzed data, and revised the manuscript. DR revised the manuscript. TLF helped with method development and manuscript revision. JL and ACC provided patient sample material and clinical information, and revised the manuscript. DFT and LAK analyzed and interpreted data, and revised the manuscript. DGB supervised the study, designed the study concept and experimental design, helped with method development and data interpretation, and revised the manuscript.

## Supplementary Material

Supplemental data

## Version 1 12/08/2020

In-Press Preview

## Version 2 01/11/2021

Electronic publication
