# Supplementary material for: Immune determinants of Barrett’s progression to esophageal adenocarcinoma
Source: JCI Insight. 2021 Jan 11;6(1):e143888. doi: 10.1172/jci.insight.143888 (PMC7821593; doi:10.1172/jci.insight.143888)
Supplement: Supplemental data [file jciinsight-6-143888-s151.pdf]

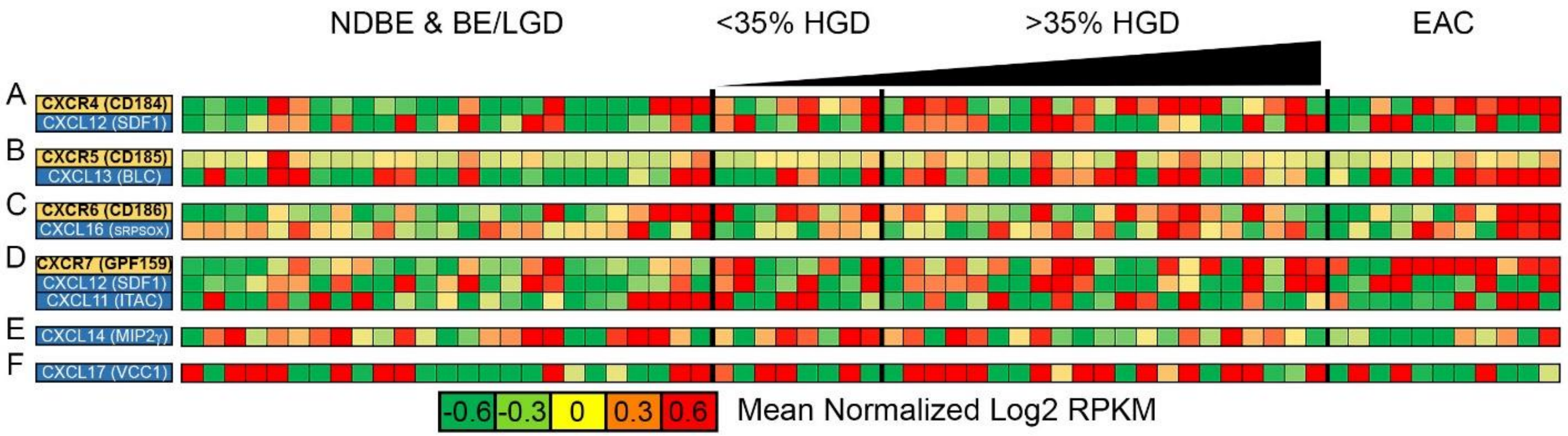

Supplemental Figure 1. CXC Expression Changes in the RNA-Seq BE progression cohort. Expression changes in a subset of CXC receptor families. There were no significant differences in the following receptor families or their associated ligands during progression from BE to EAC: CXCR4 **(A)**; CXCR5 **(B)**; CXCR6 **(C)**; CXCR7 **(D)**; CXCL14 **(E)**; and CXCL17 **(F)**. The heatmap shows mean normalized log2 (RPKM+1) expression data with the indicated color range. N=25 patients with NBDE or BE/LGD; N=8 patients with <35% HGD; N=21 patients with >35% HGD; N=11 patients with EAC. *, $p<0.05$; **, $p<0.01$; ***, $p<0.001$; as determined by Welch's t-test and Wilcoxon rank-sum tests between BE/LGD vs. HGD/EAC on log-transformed expression levels (log[RPKM + 1]).

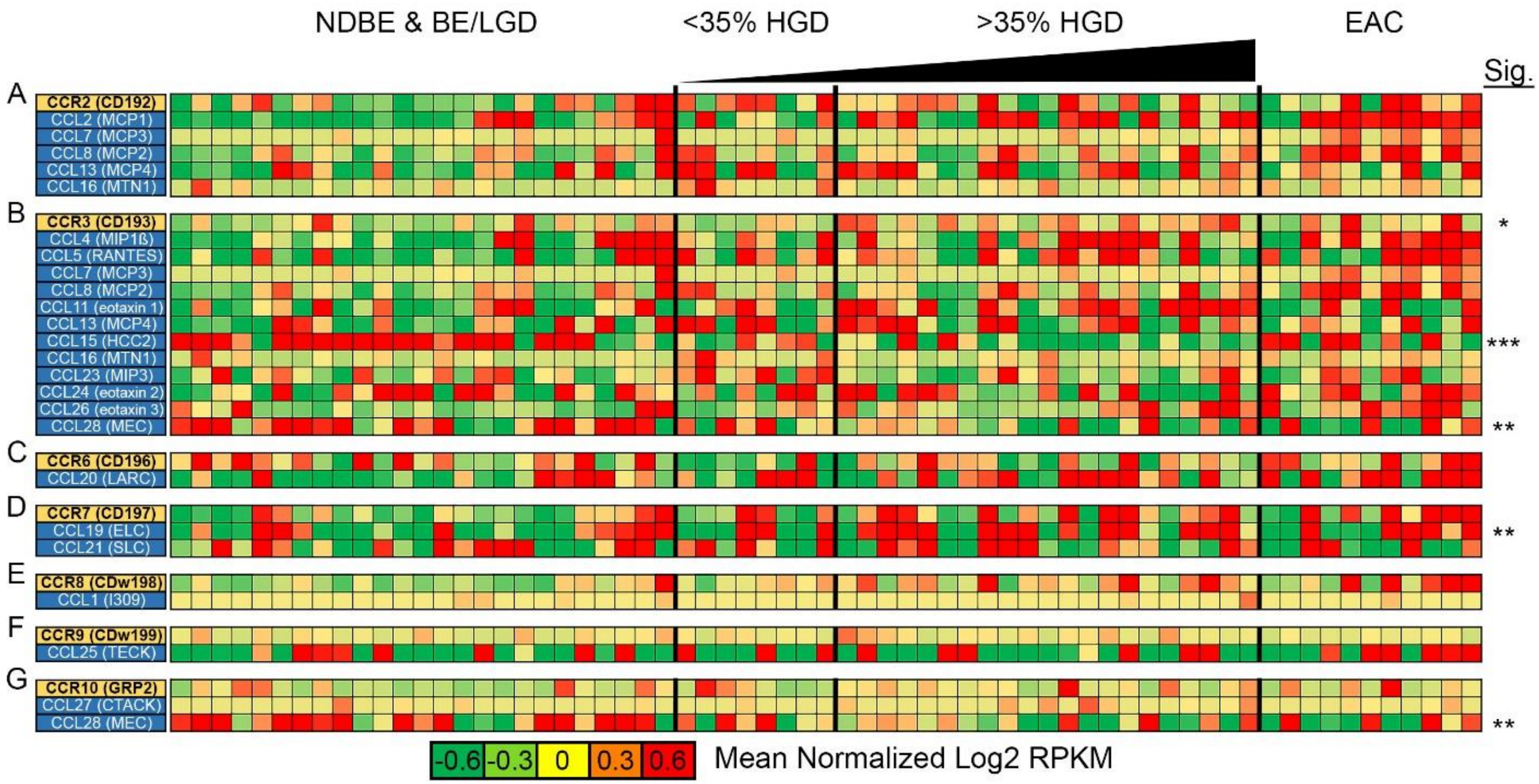

Supplemental Figure 2. CC Receptor Subfamily Expression Changes in the RNAseq BE progression cohort. Expression changes in a subset of CC receptor families. Some significant differences in select receptors/ligands (CCR3, CCL15, CCL19, CCL28) were observed during progression from BE to EAC. However, the differences were not associated with a specific receptor subfamily. The CCR subfamilies shown here are CCR2 **(A)**; CCR3 **(B)**; CCR6 **(C)**; CCR7 **(D)**; CCR8 **(E)**; CCR9 **(F)**; and CCR10 **(G)**. The heatmap shows mean normalized log2 (RPKM+1) expression data with the indicated color range. N=25 patients with NBDE or BE/LGD; N=8 patients with <35% HGD; N=21 patients with >35% HGD; N=11 patients with EAC. *, $p<0.05$; **, $p<0.01$; ***, $p<0.001$; as determined by Welch's t-test and Wilcoxon rank-sum tests between BE/LGD vs. HGD/EAC on log-transformed expression levels (log[RPKM + 1]).

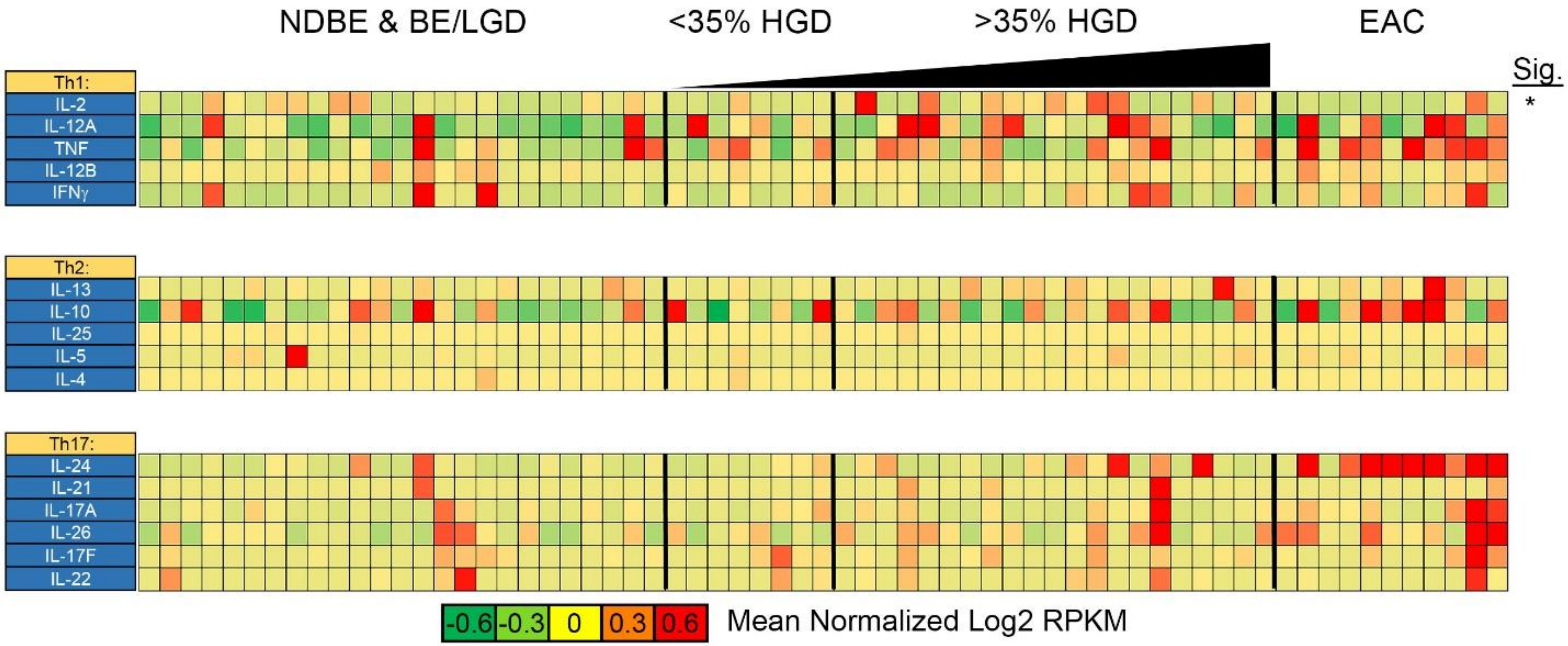

Supplemental Figure 3. T-helper Cell Gene Expression Changes in the RNAseq BE progression cohort. Gene expression changes in Th1 (top), Th2 (middle), and Th17 (bottom) gene families. There we no significant changes in gene expression in these gene families during progression from BE to EAC, outside of IL-2. The heatmap shows mean normalized log2 (RPKM+1) expression data with the indicated color range. N=25 patients with NBDE or BE/LGD; N=8 patients with <35% HGD; N=21 patients with >35% HGD; N=11 patients with EAC. *, p<0.05; **, p<0.01; ***, p<0.001; as determined by Welch's t-test and Wilcoxon rank-sum tests between BE/LGD vs. HGD/EAC on log-transformed expression levels (log[RPKM + 1]).

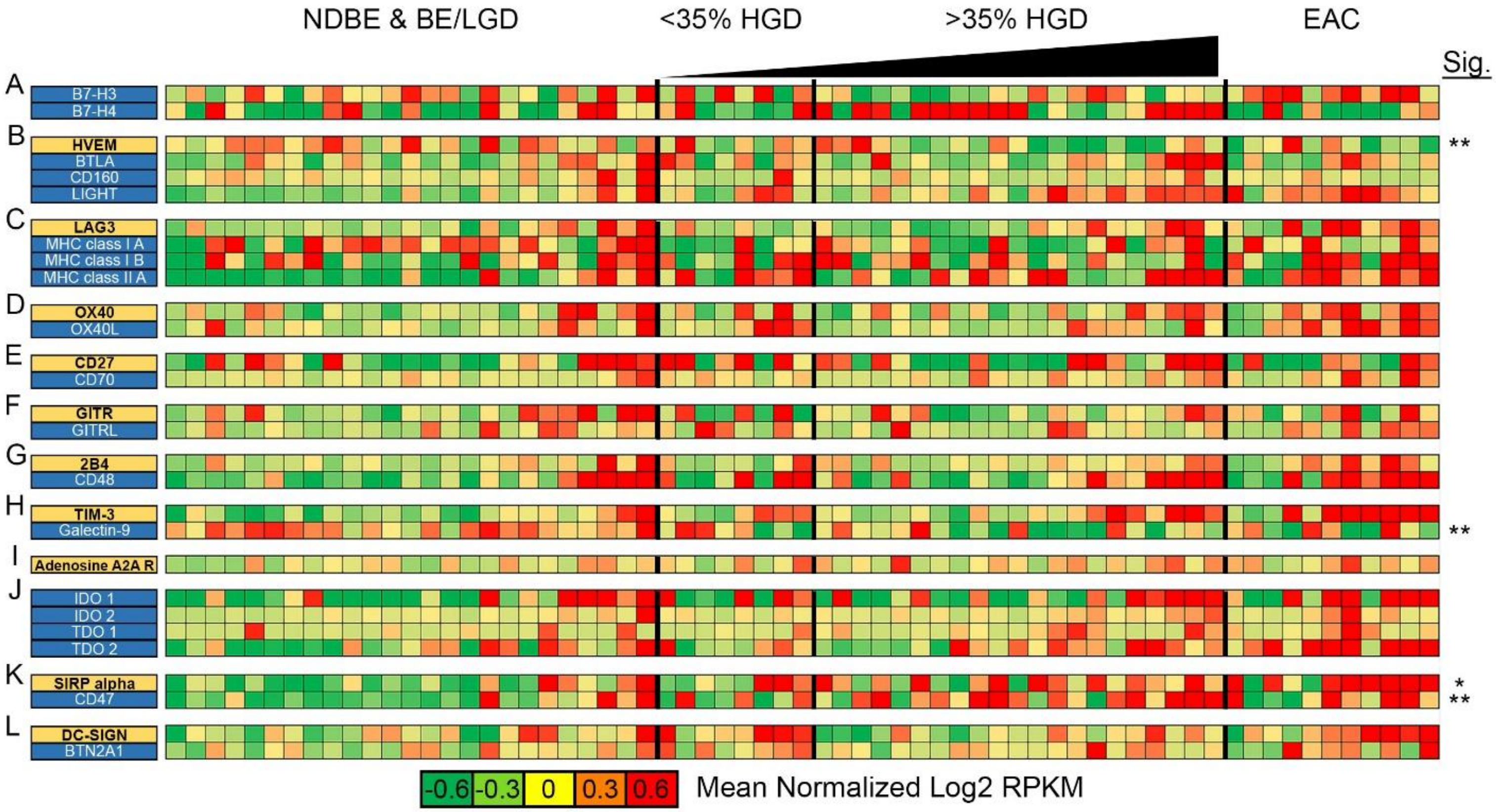

Supplemental Figure 4. Checkpoint Marker Receptor/Ligand Expression Changes in the RNAseq BE progression cohort. Expression changes in a subset of checkpoint marker receptor/ligand subfamilies. Some significant differences in select receptors/ligands (B7-H4, Galectin-9, SIRP alpha, and CD47) were observed during progression from BE to EAC. However, the differences were not associated with a specific receptor subfamily. The checkpoint marker subfamilies shown here are B7-H3/B7-H4 **(A)**; HVEM **(B)**; LAG3 **(C)**; OX40 **(D)**; CD27 **(E)**; GITR **(F)**; 2B4 **(G)**; TIM-3 **(H)**; Adenosine A2A Receptor **(I)**; IDO/TDO **(J)**; SIRP alpha **(K)**; and DC-SIGN **(L)**. The heatmap shows mean normalized log2 (RPKM+1) expression data with the indicated color range. N=25 patients with NBDE or BE/LGD; N=8 patients with <35% HGD; N=21 patients with >35% HGD; N=11 patients with EAC. *, $p<0.05$; **, $p<0.01$; ***, $p<0.001$; as determined by Welch's t-test and Wilcoxon rank-sum tests between BE/LGD vs. HGD/EAC on log-transformed expression levels (log[RPKM + 1]).

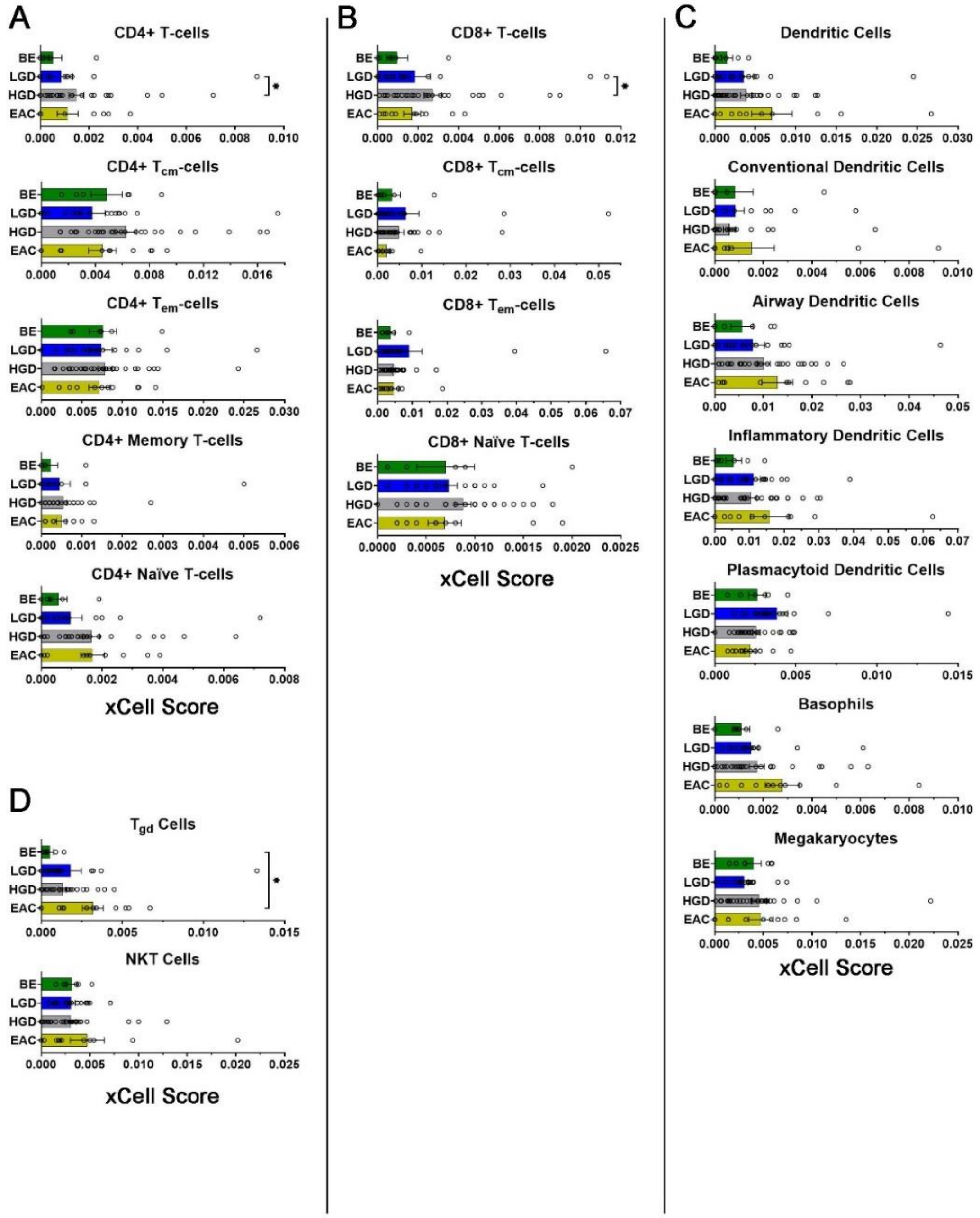

Supplemental Figure 5. xCell Analysis of Lymphocyte and Myeloid Cell Subtypes in Progression from BE to EAC. xCell Score obtained *in silico* using xCell for tissue samples from patients undergoing curative resection surgery. Histograms representing the mean value are shown along with individual data points. Analyzed lymphocyte cell type subsets include CD4+ T-cells **(A)**, CD8+ T-cells **(B)**, and NK cells **(D)**. Analyzed myeloid cell type subsets include Dendritic Cells, Basophils, and Megakaryocytes **(C)**. N=25 patients with NBDE; N=8 patients with LGD; N=21 patients with HGD; N=11 patients with EAC. *, $p<0.05$ as determined by non-parametric Kruskal-Wallis ANOVA with Dunn's correction analysis.

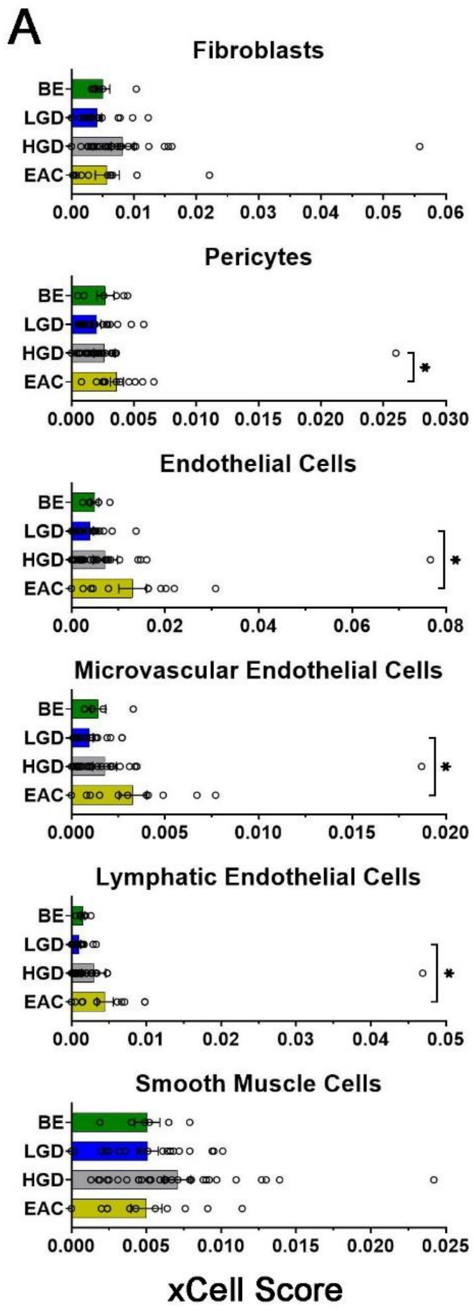

Supplemental Figure 6. xCell Analysis of Vascular Cell Types in Progression from BE to EAC. xCell Score obtained *in silico* using xCell for tissue samples from patients undergoing curative resection surgery. Histograms representing the mean value are shown along with individual data points. Analysis for vascular cell types is shown **(A).** N=25 patients with NBDE; N=8 patients with LGD; N=21 patients with HGD; N=11 patients with EAC. *, $p<0.05$ as determined by non-parametric Kruskal-Wallis ANOVA with Dunn's correction analysis.

Receptor Family	Fold-Change (BE/Low vs. HGD/EAC)	p-Value
**CXCR1 (IL-8RA)**	**3.383**	**0.000955**
**CXCL8 (IL-8)**	**4.084**	**0.016833**
**CXCL6 (GCP2)**	**4.647**	**0.037482**
**CXCL1 (GROα)**	**3.224**	**0.017477**
CXCL7 (PBP)	3.561	0.688101
**CXCR2 (IL-8RB)**	**3.402**	**0.001378**
**CXCL6 (GCP2)**	**4.647**	**0.037482**
**CXCL8 (IL-8)**	**4.084**	**0.016833**
**CXCL1 (GROα)**	**3.224**	**0.017477**
**CXCL2 (GROß)**	**2.844**	**0.000841**
**CXCL3 (GROγ)**	**2.394**	**0.00466**
CXCL5 (ENA78)	6.396	0.140597
CXCL7 (PBP)	3.561	0.688101
**CXCR3 (GPR9, CD183)**	-1.093	0.332712
CXCL13 (BLC)	1.203	0.959224
CXCL9 (MIG)	-1.541	0.45086
CXCL10 (IP10)	-1.363	0.286428
CXCL11 (ITAC)	-1.341	0.314086
CXCL4 (PF4)	1.137	0.092806
**CXCR4 (CD184)**	1.350	0.117099
CXCL12 (SDF1)	-1.048	0.169829
**CXCR5 (CD185)**	1.288	0.543074
CXCL13 (BLC)	1.203	0.959224
**CXCR6 (CD186)**	1.082	0.570965
CXCL16 (SRPSOX)	1.056	0.352541
**CXCR7 (GPF159)**	1.457	0.128822
CXCL12 (SDF1)	-1.048	0.169829
CXCL11 (ITAC)	-1.341	0.314086
CXCL14	-1.165	0.588325
CXCL17	1.089	0.209678

Supplemental Table 1. Changes in CXCR/CXCL gene expression in BE/LGD vs. HGD/EAC. Gene expression RPKM values for EAC and HGD tissue samples were averaged and compared to LGD and BE tissue samples. The ratio of this comparison is the Fold Change, where a positive fold change indicates a higher expression in HGD/EAC vs. BE/LGD and a negative fold change indicates a lower expression. p-values were generated using a Welch's t-test and Wilcoxon rank-sum test comparing BE/LGD vs. HGD/EAC, where p<0.05 is considered a significant difference (bold, right column).

Receptor Family	Fold-Change (BE/Low vs. HGD/EAC)	p-Value
CCR1 (CD191)	1.662	0.898907
CCL3 (MIP1α)	1.482	0.549357
CCL4 (MIP1ß)	1.074	0.652264
CCL5 (RANTES)	-1.340	0.426337
CCL7 (MCP3)	-1.500	0.438282
CCL8 (MCP2)	1.360	0.631162
CCL13 (MCP4)	1.273	0.181968
CCL14 (HCC1)	-1.117	0.255534
**CCL15 (HCC2)**	**-2.169**	**7.24E-06**
CCL16 (MTN1)	-1.447	0.427066
CCL23 (MIP3)	-1.187	0.815243
CCR2 (CD192)	1.145	0.253204
CCL2 (MCP1)	1.998	0.273809
CCL7 (MCP3)	-1.500	0.438282
CCL8 (MCP2)	1.360	0.631162
CCL13 (MCP4)	1.273	0.181968
CCL16 (MTN1)	-1.447	0.427066
**CCR3 (CD193)**	**1.751**	**0.038505**
CCL4 (MIP1ß)	1.074	0.652264
CCL5 (RANTES)	-1.340	0.426337
CCL7 (MCP3)	-1.500	0.438282
CCL8 (MCP2)	1.360	0.631162
CCL11 (eotaxin 1)	1.259	0.116718
CCL13 (MCP4)	1.273	0.181968
**CCL15 (HCC2)**	**-2.169**	**7.24E-06**
CCL16 (MTN1)	-1.447	0.427066
CCL23 (MIP3)	-1.187	0.815243
CCL24 (eotaxin 2)	1.315	0.444522
CCL26 (eotaxin 3)	2.410	0.117537
**CCL28 (MEC)**	**-1.371**	**0.00627**
**CCR4 (CD194)**	**1.733**	**0.029055**
CCL3 (MIP1α)	1.482	0.549357
CCL5 (RANTES)	-1.340	0.426337
CCL17 (TARC)	1.240	0.95444
CCL22 (MDC)	1.735	0.074825
CCR5 (CD195)	1.023	0.960477
CCL2 (MCP1)	1.998	0.273809
CCL3 (MIP1α)	1.482	0.549357
CCL4 (MIP1ß)	1.074	0.652264
CCL5 (RANTES)	-1.340	0.426337
CCL8 (MCP2)	1.360	0.631162
CCL11 (eotaxin 1)	1.259	0.116718
CCL13 (MCP4)	1.273	0.181968
CCL14 (HCC1)	-1.117	0.255534
CCL16 (MTN1)	-1.447	0.427066
CCR6 (CD196)	-1.051	0.102023
CCL20 (LARC)	2.365	0.433853
CCR7 (CD197)	1.817	0.195963
**CCL19 (ELC)**	**2.073**	**0.00982**
CCL21 (SLC)	-1.329	0.834304
CCR8 (CDw198)	1.765	0.406939
CCL1 (I309)	1.341	0.859157
CCR9 (CDw199)	1.145	0.251994
CCL25 (TECK)	3.197	0.919354
CCR10 (GPR2)	1.575	0.220509
CCL27 (CTACK)	2.662	0.105991
**CCL28 (MEC)**	**-1.371**	**0.00627**

Supplemental Table 2. Changes in CCR/CCL gene expression in BE/LGD vs. HGD/EAC. Gene expression RPKM values for EAC and HGD tissue samples were averaged and compared to LGD and BE tissue samples. The ratio of this comparison is the Fold Change, where a positive fold change indicates a higher expression in HGD/EAC vs. BE/LGD and a negative fold change indicates a lower expression. p-values were generated using a Welch's t-test and Wilcoxon rank-sum test comparing BE/LGD vs. HGD/EAC, where $p<0.05$ is considered a significant difference (bold, right column).

**Receptor Family**	**Fold-Change (BE/Low vs. HGD/EAC)**	**p-Value**
GM-CSF	3.090	0.4544
IL-1 Alpha	1.823	0.4639
IL-1 Beta	-1.324	0.4436
**IL-2**	**1.742**	**0.0448**
IL-3	NC	NA
IL-4	NC	0.3653
IL-5	-1.723	0.4534
**IL-6**	**3.929**	**0.0377**
**IL-7**	**-1.228**	**0.0203**
**IL-8**	**4.084**	**0.0168**
IL-10	1.247	0.7156
IL-12 Alpha	1.725	0.0864
IL-12 Beta	1.342	0.9008
**IL-15**	**-1.288**	**0.0039**
**IL-18**	**-1.297**	**0.0024**
IL-21	4.296	0.3102
IL-23 Alpha	4.181	0.7075
IL-24	17.216	0.1395
IFN Alpha1	NC	NA
IFN Alpha2	NC	NA
IFN Gamma	-1.667	0.4238
TGF Beta1	1.397	0.0761
TGF Beta2	1.448	0.2616
TNF Alpha	1.634	0.3039

Supplemental Table 3. Changes in Interleukin gene expression in BE/LGD vs. HGD/EAC. Gene expression RPKM values for EAC and HGD tissue samples were averaged and compared to LGD and BE tissue samples. The ratio of this comparison is the Fold Change, where a positive fold change indicates a higher expression in HGD/EAC vs. BE/LGD and a negative fold change indicates a lower expression. p-values were generated using a Welch's t-test and Wilcoxon rank-sum test comparing BE/LGD vs. HGD/EAC, where $p<0.05$ is considered a significant difference (bold, right column).

Receptor Family	Fold-Change (BE/Low vs. HGD/EAC)	p-Value
PD1	1.039	0.878995
PD-L1	-1.105	0.391706
PD-L2	1.084	0.645147
CD28	1.406	0.076075
CTLA4	1.556	0.308536
CD80	1.502	0.887669
CD86	1.282	0.899967
ICOS	1.605	0.450538
ICOS Ligand	1.284	0.084576
B7-H3	1.079	0.088727
B7-H4	1.284	0.062391
**B7-H7 (HHLA2)**	**-1.329**	**0.001033**
TMIGD2	1.063	0.75815
4-1BB	1.808	0.241859
4-1BBL	2.096	0.913169
**HVEM**	**-1.227**	**0.016105**
BTLA	1.206	0.213318
CD160	-1.518	0.313026
LIGHT	2.160	0.106459
LAG3	-1.137	0.416206
MHC Class I A	-1.047	0.318589
MHC Class I B	1.057	0.816099
MHC Class II A	1.228	0.215999
OX40	1.136	0.949304
OX40L	1.060	0.66258
CD27	1.110	0.345933
CD70	1.494	0.405575
CD40	1.284	0.838067
CD40L	1.074	0.518261
GITR	-1.082	0.312098
GITRL	1.071	0.895825
CD155	1.089	0.107974
DNAM-1	1.226	0.348272
TIGIT	1.343	0.541221
CD96	1.057	0.853583
2B4	1.081	0.730395
CD48	1.176	0.619044
TIM-3	1.263	0.731371
**Galectin-9**	**-1.208**	**0.001219**
Adenosine A2a R	1.443	0.069314
IDO 1	-2.456	0.410924
IDO 2	1.479	0.504833
TDO 1	2.880	0.269351
TDO 2	2.079	0.425061
**CEACAM1**	**-1.540**	**0.02155**
**SIRP alpha**	**1.516**	**0.028178**
**CD47**	**1.340**	**0.005225**
DC-SIGN	1.005	0.513818
BTN2A1	1.056	0.237536
CD200R	1.101	0.213973
CD200	1.558	0.067701
**DR3**	**1.624**	**0.000485**
**TL1A**	**1.704**	**0.000589**

Supplemental Table 4. Changes in Cytokine gene expression in BE/LGD vs. HGD/EAC. Gene expression RPKM values for EAC and HGD tissue samples were averaged and compared to LGD and BE tissue samples. The ratio of this comparison is the Fold Change, where a positive fold change indicates a higher expression in HGD/EAC vs. BE/LGD and a negative fold change indicates a lower expression. p-values were generated using a Welch's t-test and Wilcoxon rank-sum test comparing BE/LGD vs. HGD/EAC, where $p<0.05$ is considered a significant difference (bold, right column).

Receptor Family	Fold-Change (BE/Low vs. HGD/EAC)	p-Value
Th1:		
**IL2**	**1.742**	**0.044807**
IL12A	1.725	0.086392
TNF	1.634	0.303875
IL12B	1.342	0.900847
IFNG	-1.667	0.423825
Th2:		
IL13	3.176	0.204765
IL10	1.247	0.715587
IL25	-1.542	0.91989
IL5	-1.723	0.45344
IL4	NA	0.365255
Th17:		
IL24	17.216	0.139484
IL21	4.296	0.310168
IL17A	3.397	0.315551
IL26	2.156	0.136571
IL17F	1.965	0.432679
IL22	1.176	0.99788

Supplemental Table 5. Changes in T-cell family gene expression in BE/LGD vs. HGD/EAC. Gene expression RPKM values for EAC and HGD tissue samples were averaged and compared to LGD and BE tissue samples. The ratio of this comparison is the Fold Change, where a positive fold change indicates a higher expression in HGD/EAC vs. BE/LGD and a negative fold change indicates a lower expression. p-values were generated using a Welch's t-test and Wilcoxon rank-sum test comparing BE/LGD vs. HGD/EAC, where $p<0.05$ is considered a significant difference (bold, right column).

Methods:

Antibodies for Multiplex IHC Staining – Primary antibodies used for Multiplex Immunohistochemistry are as follows at the indicated dilutions. Anti-CD3 (1:400; Agilent Technologies, Catalog # A045229-2); Anti-CD8 (1:400; Spring Bio, Catalog # M5390); Anti-CD163 (1:400; Leica, Catalog # NCL-L-CD163); Anti-FoxP3 (1:400, Cell Signaling Technologies, Catalog # 12653S); Anti-PD-L1 (1:200, Cell Signaling Technologies, Catalog # 13684S); Anti-Pan-Cytokeratin (1:500, Agilent Technologies, Catalog # M351529-2). Secondary Opal antibodies and TSA Fluorophore reagents were supplied in the Opal™ 7 Solid Tumor Immunology kit and used at the recommended dilutions.
